# Physician associate (PA) students’ perceptions of team-based learning (TBL) for teaching in Geriatric medicine

**DOI:** 10.1186/s12909-025-06787-7

**Published:** 2025-02-03

**Authors:** Basaam Aweid, Natalie Parnis, David Harrison

**Affiliations:** 1 Elderly Day Hospital, The Hillingdon Hospitals NHS Foundation Trust, Pield Heath Road, Uxbridge, UB8 3NN UK; 2 College of Health, Medicine and Life Sciences, Brunel University London, Uxbridge, UB8 3PH UK; 3 Education Department, Royal College of Physicians, 11 St Andrews Place, Regent’s Park, London, NW1 4LE UK

**Keywords:** Physician associate, Physician assistants, Team-based learning, Geriatrics

## Abstract

**Background:**

Physician Associates have recently been introduced to the UK healthcare workforce. Their curriculum includes important topics in Geriatric medicine. As they undertake a 2-year intensive postgraduate course we wanted to explore if Team-based Learning is an effective and efficient learning strategy. In addition, we wanted to know how this approach compares to their current learning methods, namely Problem-based learning and lectures.

**Methods:**

This was a qualitative study of Physician associate student perceptions of Team-Based Learning. After introducing four TBL sessions in the specialty of Geriatric medicine we sent students anonymous questionnaires asking them about their TBL experience and how it compared to PBL and lectures. We then undertook a thematic analysis of the responses.

**Results:**

Twenty students responded to our online questionnaire. The thematic analysis utilised themes from previous studies as well as emergent ones. The key themes were that TBL requires *more preparation*, *TBL is effective*, TBL is *preferred to PBL and lectures*, but students *did not want TBL to replace all their teaching*.

**Conclusions:**

TBL is an effective learning strategy that can be used alongside other teaching methods. While Physician Associate students preferred TBL over PBL and lectures, they wanted to have TBL alongside lectures at least. This information is important when planning teaching for an intensive 2-year course.

**Supplementary Information:**

The online version contains supplementary material available at 10.1186/s12909-025-06787-7.

## Background

### Physician associates

Physician Associates (PAs) were introduced in the UK in 2003. In the USA they are called Physician *assistants* where they have been present since the 1960s [1]. They are defined as “Healthcare professionals who work under the supervision of a doctor within multi-disciplinary teams” [2]. At Brunel University London, we have been running the PA programme as a 2-year, postgraduate Masters course since 2016. Students must have an undergraduate degree in a health science in order to apply to PA courses in the UK.

Since December 2024, The General Medical Council (GMC) has become the regulating body for PAs in the UK [2]. After qualifying from a University PA course, students are expected to complete national OSCE (Objective Structured Clinical Exam) and written exams before being able to practice. PAs at Brunel University London (BUL) are currently taught through Problem-based learning (PBL), Case-based learning and lecture-based learning. Our university has recently introduced a medical school with their first intake in 2022. This is a unique MBBS course with the majority of teaching delivered using Team-Based Learning. We wanted to assess TBL as a teaching method for PA students.

### Team-based learning

Team-based learning is a learning strategy first developed by Larry Michaelsen in the 1970s [3]. It can be summarised into the following 3 stages:

1. Preparation: Preparatory material is sent to students before the session. This can include recorded lectures, chapters in a book or guidelines. Students are required to study this before coming to the session.
2. Readiness Assurance Tests: In this process, students undertake an individual test called the individual readiness assurance test (iRAT). This is then followed by the students undertaking the same test but within their team of 6–8 peers. This is called the team readiness assurance test (tRAT). At this stage, the correct answer to each question is revealed to the ‘team leader’ and students discuss further submitting their selections until they arrive at the correct answer.
3. Application Exercise: Each team is then given new practical questions to solve by applying the knowledge they have gained from the first 2 stages. These questions usually consist of real-life clinical scenarios.

**Fig. 1:**
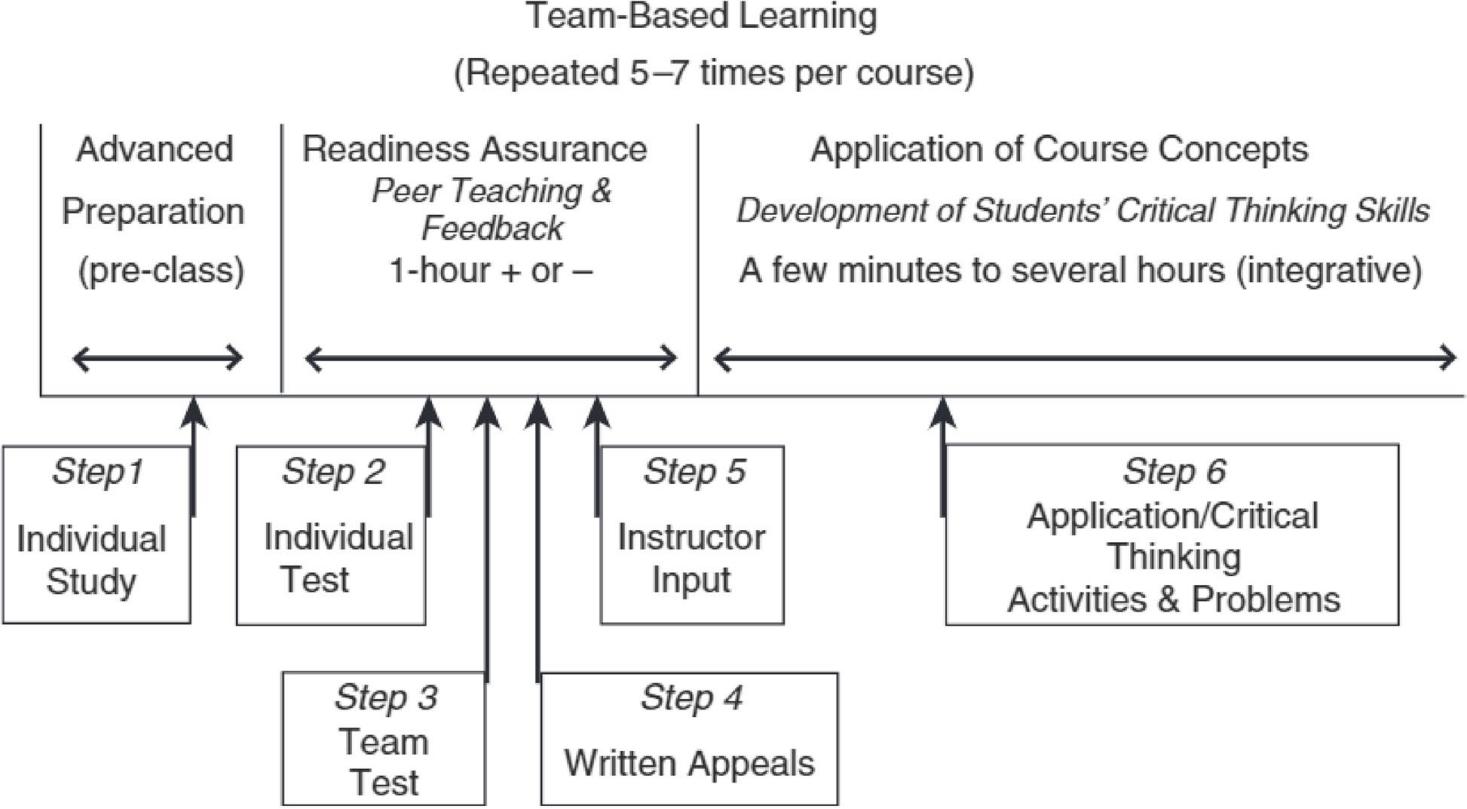
Instructional activity sequence for TBL content unit [4]

This process, with further steps within each stage, is summarised in Fig. 1.

### TBL and PA education

The authors did not find any specific literature outlining the type of teaching available to PA students at different universities. However, from our experience with the PA Schools Council (PASC) we know that this consists mainly of lecture-based teaching and Problem-based Learning (PBL). Our students have a PBL session once a week. The rest of their teaching is lecture-based or practical clinical skills teaching. At the largest PA course in the UK, the main teaching methods include lectures, problem-based learning (PBL), and self-directed learning [5].

A literature search identified only 4 studies that specifically explored TBL and PA students [6–9]. All these studies took place in the USA with no UK studies. One of these studies had a mixed sample of PA and nursing anaesthetic students [7]. All these articles looked at small samples of PA students ranging from 27 to 67 students in each study. Additionally, one of our authors recently published an abstract reviewing the first TBL session in a UK PA programme in a small sample of 17 PA students [10]. These studies all ranged from being purely qualitative exploring student perceptions to mixed studies that also compared exam performance between PA cohorts taught with or without TBL. The results of these studies can be divided into those looking at student performance and those exploring student perceptions.

#### TBL and PA student’s performance

Isbell et al. [7], Nguyen et al. [6] and Patel et al. [9] evaluated TBL for teaching gross anatomy, clinical pharmacology and Paediatric preventative medicine, respectively. Table 1 summarises these studies.

**Table 1:** Summary of quantitative performance studies that measured the effectiveness of TBL through its impact on the PA students’ test results

Study component	Isbell et al. [7]	Nguyen et al. [6]	Patel et al. [9]
No. of students	*N* = 93; 56PA, 37NA (control) *N* = 98; 67PA, 31NA (TBL + practical)	*N* = 36 (control) *N* = 35 (TBL + lectures)	No control *N* = 27 (TBL only)
Type of study	Case–control	Case–control	Cohort
Teaching in control group	Practical only	Lectures only	N/A
Place of Study	USA	USA	USA
Subject taught	Gross Anatomy	Clinical Pharmacology	Paediatric Nutrition
Outcome measure	Practical and written exams	Summative exam results	Specific Pre and post-TBL course test
Performance Results	*TBL group better* *(p* < *0.05)*	*No significant difference (p* = *0.24)*	*Improved score after TBL (p* < *0.05)*
Other results	N/A	Better student perception after TBL	Better student perception after TBL

Isbell et al. [7] investigated a cohort of PA and nurse anaesthetist (NA) students. They found that the 2014 cohort who were taught using TBL performed significantly better in written and practical examinations compared to their 2013 cohort who were taught through traditional lectures and laboratory teaching only (*p* < 0.05).

Nguyen et al. [6] compared their 2013 PA student cohort (*n* = 36) who were taught by lectures only, with their 2014 PA student cohort (*n* = 35) who were taught by a combination of lectures and TBL. They found no significant difference between the two groups in their summative exam scores (*p* = 0.24). However, this study was limited as it excluded the 3rd application phase of TBL which it can be argued is essential as it resembles clinical practice [6].

Patel et al. [9] evaluated TBL in teaching a new Paediatric subject in their course entitled ‘Nutrition and Preventative Medicine across the lifespan’. They found a significant improvement in the test results of the students after TBL (*p* < 0.05). This simply showed that TBL improved students’ knowledge after the teaching session. There was no control group.

An additional study by Loftin and West [8] conducted a self-efficacy survey pre-intervention and post-intervention on 87 PA students who were randomised to either TBL or non-TBL learning (via online modules). Their outcome measure was ‘self-efficacy’ (confidence) and they found areas of increased confidence in the student group taught using TBL compared to the control group who learned through online modules (*p* < 0.05).

#### TBL and PA students’ perceptions

Two of the above performance studies were mixed and looked at qualitative data in addition to the performance measures. Nguyen et al. [6] and Patel et al. [9] used similar themes in their student feedback questions post-TBL teaching to evaluate student perceptions. The results are summarised in Table 2.

**Table 2:** PA student feedback from two studies that explored similar themes around student perceptions of TBL

Student Feedback (On post-TBL survey)	Nguyen et al. [6] (*N* = 33)	Patel et al. [9] (*N* = 20)
Previous exposure to TBL	73%	45%
Prepared for TBL session (pre-class)	75%	85%
Preferred TBL over lectures	48%	65%
TBL is more effective at retaining information	61%	75%
TBL assessments improved in-class learning	85%	100%

In general, most students had a positive perception of TBL in the context of the studies. Feedback around lack of preparation time is important and relevant to an intensive PA course. It highlights that the introduction of TBL in addition to its preparation time can be challenging. We found similar outcomes in our evaluation of a single TBL session on Stroke medicine [10].

To add to the above data, we wanted to explore student perceptions of TBL delivered over an entire module within our UK PA programme.

### Geriatric medicine

Geriatric medicine or elderly care encompasses a range of chronic conditions that are covered within different specialities of our foundations of clinical medicine study block [11]. The topics that are taught each year through lectures are as follows:

1. Geriatric Giants
2. Delirium and the Dementias
3. Stroke medicine
4. Frailty
5. Falls

We felt that this specialty combines teaching from multiple areas of clinical medicine. It therefore lends itself well to team-based learning with clinical application exercises. Therefore, for our 2023 cohort, we decided to deliver these topics through 4 TBL sessions and explore students’ perceptions of this teaching method.

## Methods

This was a qualitative study using constructivist grounded theory to collect and analyse data on 1st year PA students’ perceptions of TBL after 4 sessions within the speciality of Geriatric medicine. These sessions took place over the first academic year of the PA programme (2023). They were delivered face-to-face in a classroom setting with round tables; a set up conducive to team activities. There was a TBL facilitator and a content expert in each session. Each TBL session lasted 3 h which is the same length of time that the lectures on these topics would have taken. At the time, The College of Health, Medicine and Life Sciences (CHMLS) was keen for programmes to use innovative approaches to teaching therefore the programme team were supportive of this pilot. TBL was already considered an acceptable option to teach medical topics.

The class was split up into teams of 4. Each team had 6–7 students and the teams remained the same for all sessions. To facilitate collaboration, these teams were the same teams that the students were assigned to for their PBL sessions that they had once a week. The 4 TBLs were delivered over a course of 4 months and this overlapped between their 2nd and 3rd term of their first PA academic year.

We adhered to the 3 stages of TBL as described by Michaelsen and Sweet [3]. For our specific cohort this was done as follows:

1. Preparation material was sent to students at least 1 week before their TBL session. This included reading material in textbooks, guidelines and lectures. This was expected to take 2–3 h of a student’s time. They had half a day of timetabled weekly ‘self-directed learning’ to accommodate this.
2. Students then started the session with an individual test, the individual readiness assurance test (iRAT). Students were given between 15–20 min to complete a set of Single Best Answer (SBA) questions. We used 15–20 SBAs in each of our TBL sessions. They then undertook the same test in collaboration with their group, the team readiness assurance test (tRAT). They were given 20 min to complete the answers. The team leader then submitted the final group answer for each question. This section was a ‘closed book’ exercise therefore students relied on each other’s preparation only and could not access other resources. The iRAT and tRAT questions were all formatted as SBA questions.
3. Finally, each team completed an application exercise (AE), that was reflective of real-life clinical cases. The AEs ranged from videos of a patient consultation to cases with investigation results such as X-rays, bloods and CT or MRI scans. These questions were a mixture of SBAs, short answer questions and gallery walks. Gallery walks allowed teams to review the work of their peers, provide constructive feedback and star rate other teams’ results [12].

The AE questions are particularly important for a successful TBL. We made sure they adhered to Parmelee & Michaelsen’s 4 Ss [4]:

1. Significant “real life” clinical case
2. Same case used for all the teams
3. Specific choices to answer specific questions
4. Simultaneously reporting from the teams

We used the LAMS (learning activity management system) application to conduct all the TBL sessions. This was an effective online platform that allowed us to monitor student progress and results in real time. This also allowed for immediate digital feedback of the answers during the tRAT exercise. Traditionally this was done using scratch cards for TBL as ‘immediate feedback’ is another core element of TBL.

None of the scores from the iRAT, tRAT and AE were used for any summative assessment. However, the format of the iRAT and tRATs was similar to their summative written exam questions. It was hoped that the focus would be on learning rather than assessment.

To evaluate perceptions, our 24 1st year students were then sent an anonymous online survey after their 4th and final TBL session that took place in term 3 of their first year (2023).

Students were informed that the purpose of these surveys was to evaluate TBL as a new learning strategy that may be used further for their teaching in the second academic year as well as for future PA cohorts. It was made clear that the survey was voluntary and anonymous with no impact on their marks.

To allow for comparability between the studies, we based our questionnaire on the themes covered by Nguyen et al. [6] and Patel et al. [9] shown in Table 2. The themes assessed included preparation, effectiveness, and group discussions. In addition to these past themes, we included specific questions comparing TBL to other teaching methods such as PBL and lectures. We felt that this was particularly important information for an intensive 2-year programme that may require the selection of one teaching method in preference to another. Comparing TBL to PBL has also never been done in the past studies involving PA students.

We used a mixture of Likert scales and white-space questions for the questionnaire. As this is a small study with only 24 students in the first year, we were keen to focus on the richness of the qualitative data. This survey was developed specifically for this study (Supplementary material, Figure S1).

A brief thematic analysis of the anonymous data was then undertaken, to identify patterns in student responses [13]. The questionnaire analysis used a priori codes identified from the 4 aforementioned studies involving PAs and TBL [6, 9]. However, given the small sample size, attempts to capture and analyse all emerging themes would be limited if only a priori coding was applied, so as new information was sought, we used a combination of a priori and emergent coding.

## Results

**Fig. 2:**
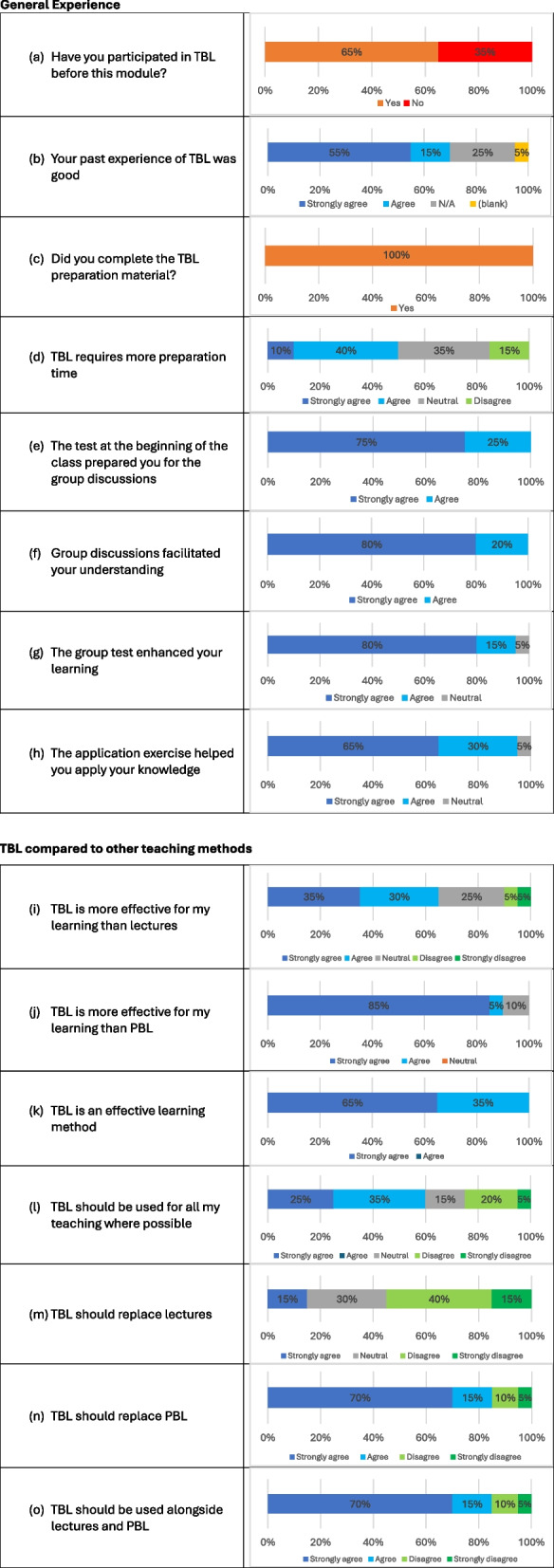
Geriatic TBL feedback results (*n* = 20)

A total of 20 students responded to the questionnaire (83% response rate). The results are shown in Fig. 2. It was observed that 13 out of the 20 (65%) respondents had previously participated in TBL before this module (Fig. 2a) with a majority of these (70%) indicating that their past experience of TBL was good (Fig. 2b). Students made some interesting comments about their past experience including “*being able to work in a team and learning better than PBL*”.

While all students (100%) stated that they completed the preparation material before class (Fig. 2c), half of them (50%) commented that TBL needs more preparation time (Fig. 2d).

When students were asked about their Geriatric TBL module experience, all respondents felt that the iRAT *prepared them for the group discussion,* with 75% of them strongly agreeing with this statement (Fig. 2e). All students agreed or strongly agreed that the *group discussions facilitated their understanding of the topic* (Fig. 2f). Apart from 1 student they all felt that the *tRAT enhanced their learning*; 80% of them strongly agreed with this statement as shown in Fig. 2g. All students, apart from 1 (who was neutral) agreed or strongly agreed that *the application exercise helped* them *apply* their *knowledge* (Fig. 2h).

Moreover, the feedback in comparing TBL to other teaching methods showed that the majority of students (85%) strongly agreed that *TBL is more effective for* their *learning than PBL* (Fig. 2j). When asked for their opinion on whether *TBL is more effective for their learning than lectures, 65% of* respondents strongly agreed or agreed with this statement (Fig. 2i).

The students’ overall TBL experiences indicated that 65% of students strongly agreed that *TBL is an effective learning method* (Fig. 2k)*.* While most students believed that *TBL is more effective than lectures*, only 15% of students said that *TBL should replace lectures* (Fig. 2m) with 55% disagreeing with this statement (40% strongly disagreeing). However, 85% of respondents stated that *TBL should replace PBL,* with 70% strongly feeling this way (Fig. 2n)*.*

55% of respondents were in favour of having *TBL for all their teaching*; with only 25% strongly agreeing with this statement (Fig. 2l). Most students (85%) were in favour of using TBL *alongside lectures and PBL* with 55% strongly agreeing with this statement (Fig. 2o).

### Thematic analysis of results

As explained, we used some of the themes covered in previous studies to generate our survey to allow comparability. Table 3 shows how our data compares with the themes explored by Nguyen et al. [6] and Patel et al. [9].

**Table 3:** BUL study results compared to previous studies from Table 2

Student Feedback (On post-TBL survey)	Nguyen et al. [6] (*N* = 33)	Patel et al. [9] (*N* = 20)	Our BUL Survey (*N* = 20)
Previous exposure to TBL	73%	45%	65%
Prepared for TBL session (pre-class)	75%	85%	100%
Preferred TBL over lectures	48%	65%	65%
TBL is more effective at retaining information	61%	75%	N/a
TBL assessments improved in-class learning	85%	100%	95%

The comparative data in Table 3 illustrates that BUL results are consistent with Nguyen et al. [6] and Patel et al. [9] studies regarding students’ perception that engaging in TBL assessments improves their learning. It is also evident that students were committed to preparing for TBL sessions in all three studies. We can also note that BUL results for students’ preference of TBL over lectures is similar to the finding by Patel et al. [9].

In addition to themes from previous studies, we also asked questions focused on comparing TBL to other teaching methods. Table 4 summarises these results.

**Table 4:** Comparing TBL to other teaching methods

Student Perception Themes [% who agree or strongly agree]	BUL Survey 2023
TBL should replace PBL	85%
TBL should replace lectures	15%
TBL use for all teaching	60%
TBL requires more preparation	50%
TBL use alongside other methods	90%

Students’ comments reinforced the data from the survey that although TBL is good for students’ learning, they would like to have lectures alongside TBL sessions. Some students thought that ‘a*lthough TBL is extremely informative and keeps* [them] *engaged on specific topics’,* they believed it should not replace lectures. They explained that TBL would be most beneficial for learning complex topics (e.g., COPD, asthma, endocrine thyroid, diabetes). According to students, having lectures alongside TBL would ensure that students ‘*are taught more holistically about a particular topic’*.

## Discussion

This was a qualitative evaluation study of the perception of TBL for teaching Geriatrics in a UK PA programme. It is a unique study comparing TBL to both PBL and lectures in PA education. It is also the 5th study that evaluated the use of TBL in PA students. Unlike previous studies, our focus was on student perceptions rather than objective performance in exams. This was important as a first stage in the implementation of a new teaching method. We wanted to know if students preferred TBL to the current teaching methods before investing in the resources required to deliver TBL.

Our study is consistent with previous studies [6, 9] where students felt TBL improved classroom learning. Most students preferred TBL over lectures in our study as well as the study by Patel et al. [9]. Nguyen et al. [6] found that only 48% of students preferred TBL over lectures. In our study we wanted to explore the implications of this further and identify if students wanted TBL to replace all other teaching as well as comparing it to PBL. While initially 60% of students felt that TBL should be used for all teaching, when probed further with another option, the majority (90%) of students wanted TBL to be used alongside other teaching methods. If given the option to choose one, 85% of students wanted TBL to replace PBL and 65% preferred TBL over lectures.

These results show a consensus but also identify variability between students. While the majority feel that TBL improves learning in the classroom (95%), there is a smaller majority (60%) that feel TBL should replace all teaching. This indicates that TBL is an effective teaching method, at least as effective as PBL and lectures, but serves as an additional supplementary teaching method. Where delivering multiple teaching methods may be challenging, our results suggest that TBL could be used to replace PBL but not to replace lectures.

We could find one study that explored such a combined teaching approach by adding a TBL session to the end of 3 PBL sessions in a novel ‘package approach’ [14]. Through questionnaires exploring students perceptions, they found that their medical students valued this TBL-PBL combination.

We found only one study that directly compared PBL to TBL. This explored PBL vs. TBL in 1st year medical students [15]. Their findings corroborated our results where students overwhelmingly preferred TBL over PBL.

### Limitations

One of the questions in our evaluation asked if students had experienced TBL before. As this questionnaire was conducted at the end of 4 TBL sessions, some students assumed that previous experience included the sessions they had just completed even though our question stressed the words “before this module”. In addition, some students interpreted TBL as any form of ‘team learning’.

Like previous studies of TBL in PA education, our sample was small (n = 20). Our study, similar to the US studies, looked at TBL in only 1 module or specialty. Despite this, we were able to derive some rich data. However, it would be more informative to explore TBL over a whole year with different subjects.

As with previous studies, our study explored TBL as a new approach resulting in potential bias from students due to the *novelty effect* [16]. It is important to look at the longer-term effect of TBL to see if these positive perceptions persist.

We explored TBL in 1st year PA students. It would be interesting to see if these findings are replicated in second year PA students where the teaching is more clinically focused with students working in hospital departments within teams. This may lend itself better to TBL.

Unlike previous studies, we focused on qualitative findings. A study exploring the effect of TBL on student’s exam performance may provide more objective and quantitative results to see if there is alignment with our qualitative findings.

### Future research

This study needs to be replicated in larger samples of PA students over the whole PA curriculum. Given our results, it would be incumbent to specifically explore TBL compared to other teaching methods to see if our results are generalisable. TBL not only places greater onus on the student, in terms of preparation, but also adds a teaching load on the academic staff. The preparation material, in-class tests and application exercises require a significant amount of careful planning and additional work not normally needed with traditional lectures. A study to explore academic staff perceptions of TBL would provide a more holistic understanding of the effectiveness and implementation of TBL.

## Conclusion

Introducing TBL alongside lectures is a teaching strategy favoured by students in PA education. As most courses already use PBL, an acceptable alternative would be to replace some PBL with TBL.

Our data is consistent with previous results indicating that TBL requires more preparation than other teaching methods. Any teaching programme would need to consider this carefully when planning their students’ timetable. This is particularly important in an intensive 2-year course such as a PA programme.

## Supplementary Information

Supplementary Material 1.

Supplementary Material 2.

## Data Availability

The survey results are provided within the supplementary material section (Supplementary material, Figure S2).

## Abbreviations

PA: Physician Associate or Assistant
BUL: Brunel University London
TBL: Team-based learning
iRAT: Individual readiness assurance test
tRAT: Team readiness assurance test
AE: Application exercise
PBL: Problem based learning
LAMS: Learning activity management system
