# Supplementary material for: Physician associate (PA) students’ perceptions of team-based learning (TBL) for teaching in Geriatric medicine
Source: BMC Med Educ. 2025 Feb 3;25:173. doi: 10.1186/s12909-025-06787-7 (PMC11792404; doi:10.1186/s12909-025-06787-7)
Supplement: Supplementary file 1 — Supplementary Material 1. [file 12909_2025_6787_MOESM1_ESM.pdf]

Geriatric TBL Survey

Page 1: Geriatric TBL module feedback

1. Have you participated in TBL before this module?

- Yes
- No

2. Past experience. Please indicate your level of agreement

Please don't select more than 1 answer(s) per row.

	Strongly agree	Agree	Neutral	Disagree	Strongly disagree	N/A
Your past experience of TBL was good	☐	☐	☐	☐	☐	☐

2.a. Please comment on your answer

3. Did you complete the TBL preparation material?

- Yes
- No

3.a. Please comment on your answer

4. Geriatric TBL module experience. Please indicate your level of agreement.

Please don't select more than 1 answer(s) per row.

	Strongly agree	Agree	Neutral	Disagree	Strongly disagree
TBL requires more preparation time	☐	☐	☐	☐	☐
The test at the beginning of the class prepared you for the group discussions	☐	☐	☐	☐	☐
Group discussions facilitated your understanding	☐	☐	☐	☐	☐
The group test enhanced your learning	☐	☐	☐	☐	☐
The application exercise helped you apply your knowledge	☐	☐	☐	☐	☐

5. TBL compared to other teaching methods. Please indicate your level of agreement

Please don't select more than 1 answer(s) per row.

	Strong agree	Agree	Neutral	Disagree	Strongly disagree

TBL is more effective for my learning than lectures	☐	☐	☐	☐	☐
TBL is more effective for my learning than PBL	☐	☐	☐	☐	☐

5.a. Please comment on your answer

6. Overall experience. Please indicate your level of agreement

Please don't select more than 1 answer(s) per row.

	Strongly agree	Agree	Neutral	Disagree	Strongly disagree
TBL is an effective learning method	☐	☐	☐	☐	☐
TBL should be used for all my teaching where possible	☐	☐	☐	☐	☐
TBL should replace lectures	☐	☐	☐	☐	☐
TBL should replace PBL	☐	☐	☐	☐	☐
TBL should be used alongside lectures and PBL	☐	☐	☐	☐	☐

6.a. Please comment on your answer

7. Do you have any other comments?

Page 2: Final page
