# Supplementary material for: Physician associate (PA) students’ perceptions of team-based learning (TBL) for teaching in Geriatric medicine
Source: BMC Med Educ. 2025 Feb 3;25:173. doi: 10.1186/s12909-025-06787-7 (PMC11792404; doi:10.1186/s12909-025-06787-7)
Supplement: Supplementary file 2 — Supplementary Material 2. [file 12909_2025_6787_MOESM2_ESM.pdf]

Geriatric TBL Survey (PA students)

Showing 20 of 20 responses

Showing **all** responses

Showing **all** questions

Response rate: 20%

1 I have read the participant information included with this questionnaire

YES	**20** (100%)
NO	**0**

1.a I am over the age of 18

YES	**20** (100%)
NO	**0**

1.b I understand that no personal identifying data is collected in this study, therefore I know that once I have completed the questionnaire I am unable to withdraw my data from the study

YES	**20** (100%)
NO	**0**

1.c I agree that my data can be anonymised, stored and used in future research in line with Brunel University's data retention policies

YES	**20** (100%)
NO	**0**

1.d I agree that all opinions expressed in the questionnaire will be kept confidential.

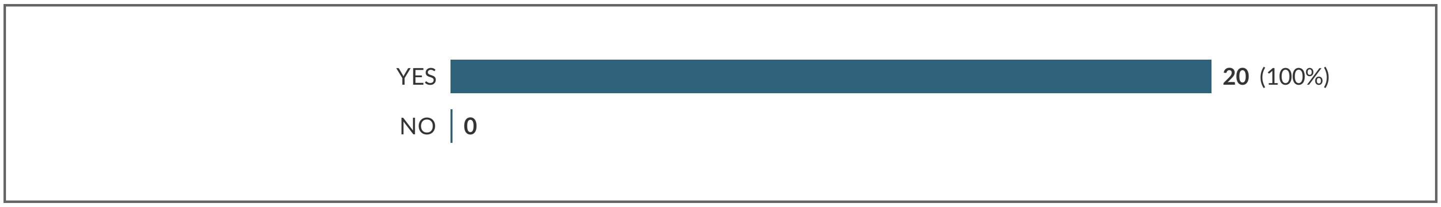

1.e I agree to take part in this study

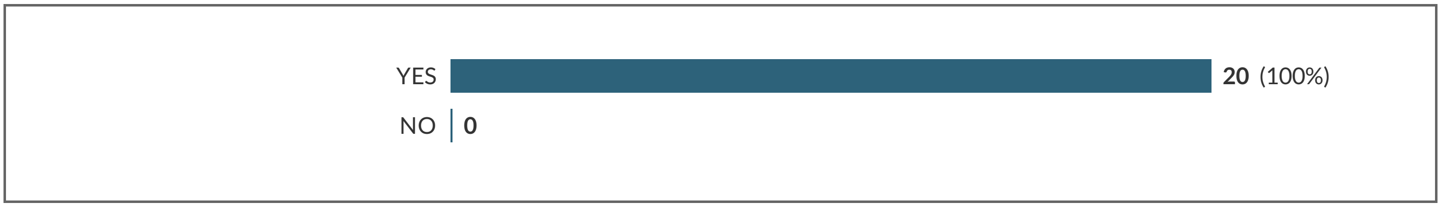

2 Have you participated in TBL before this module?

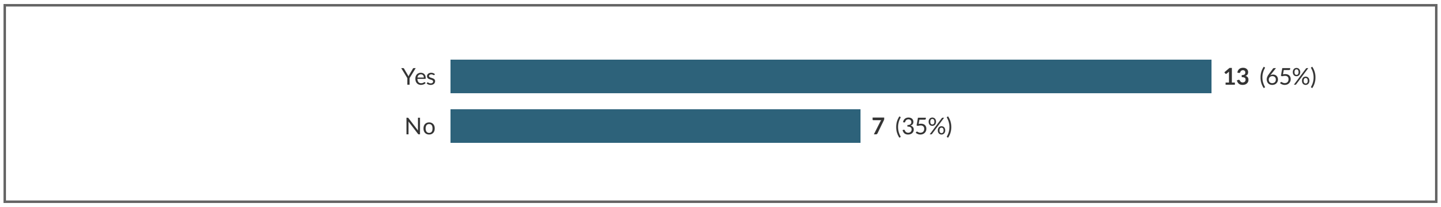

3 Past experience. Please indicate your level of agreement

3.1 Your past experience of TBL was good

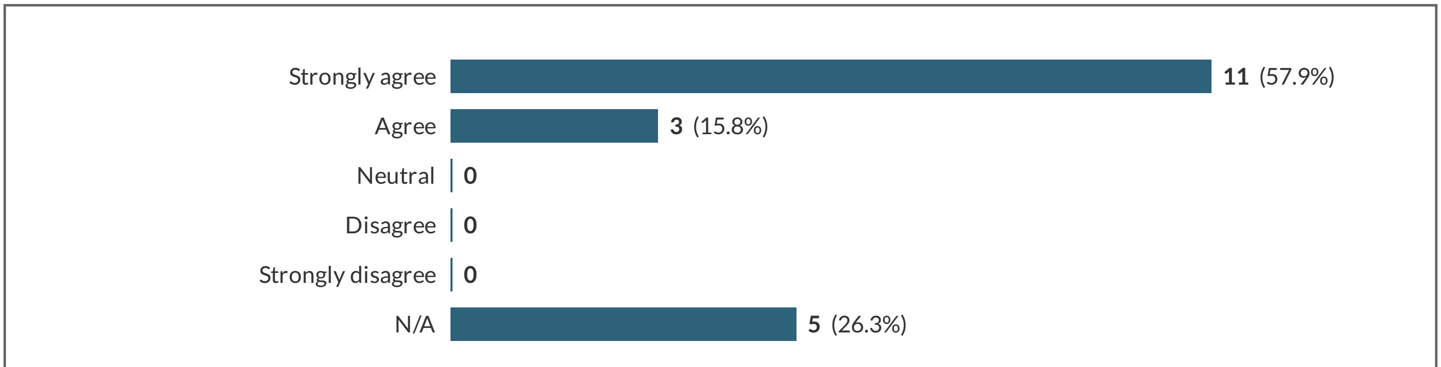

Multi answer: Percentage of respondents who selected each answer option (e.g. 100% would represent that all this question's respondents chose that option)

3.a Please comment on your answer

Showing first 5 of 15 responses	
I like that the TBL has an interactive componenet	1082499-1082481-114082304
Have never previously participated in TBL, this module was first introduced in this course and it is extremely useful	1082499-1082481-114082208
its always been engaging and informative	1082499-1082481-114082256
Very interactive and taught me how to argue points	1082499-1082481-114082223
able to work in a team and learning better than PBL	1082499-1082481-114082346

4 Did you complete the TBL preparation material?

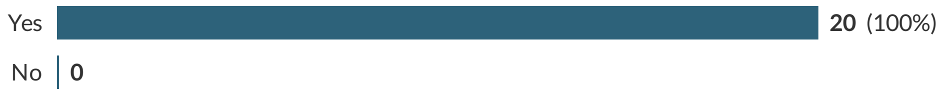

4.a Please comment on your answer

Showing first 5 of 10 responses	
the preperation material really helps us to deepen our knowledge before.	1082499-1082481-114082304
yes i did	1082499-1082481-114082208
Completed	1082499-1082481-114082256
when material is provided, preparation is always done beforehand	1082499-1082481-114082281
The before hand information was helpful	1082499-1082481-114082227

5 Geriatric TBL module experience. Please indicate your level of agreement.

5.1 TBL requires more preparation time

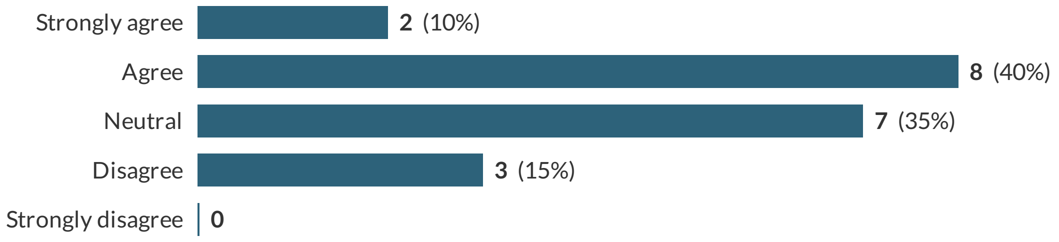

Multi answer: Percentage of respondents who selected each answer option (e.g. 100% would

represent that all this question's respondents chose that option)*

5.2 The test at the beginning of the class prepared you for the group discussions

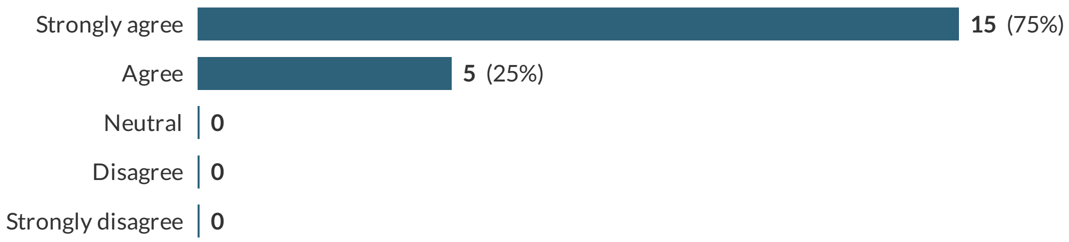

Multi answer: Percentage of respondents who selected each answer option (e.g. 100% would represent that all this question's respondents chose that option)

5.3 Group discussions facilitated your understanding

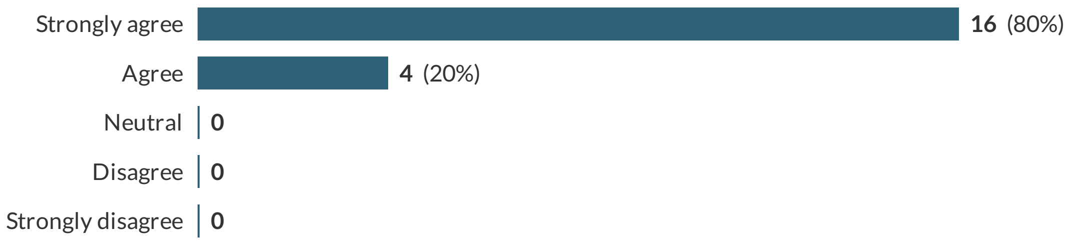

Multi answer: Percentage of respondents who selected each answer option (e.g. 100% would represent that all this question's respondents chose that option)

5.4 The group test enhanced your learning

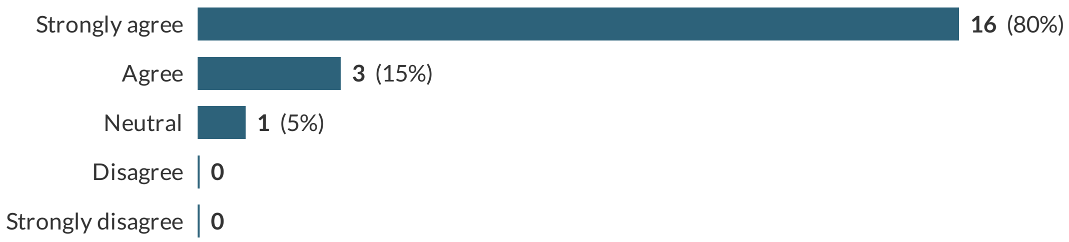

Multi answer: Percentage of respondents who selected each answer option (e.g. 100% would represent that all this question's respondents chose that option)

5.5 The application exercise helped you apply your knowledge

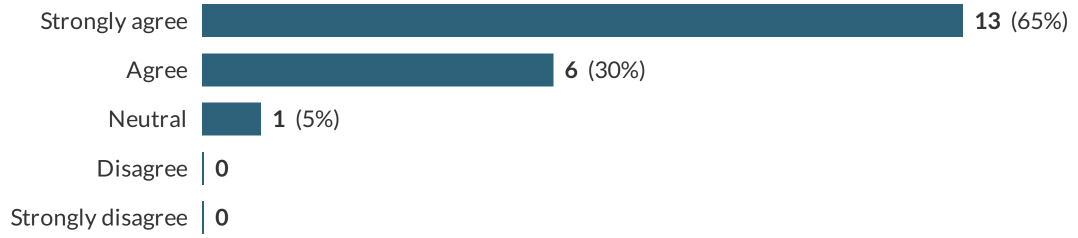

Multi answer: Percentage of respondents who selected each answer option (e.g. 100% would represent that all this question's respondents chose that option)

6 TBL compared to other teaching methods. Please indicate your level of agreement.

6.1 TBL is more effective for my learning than lectures

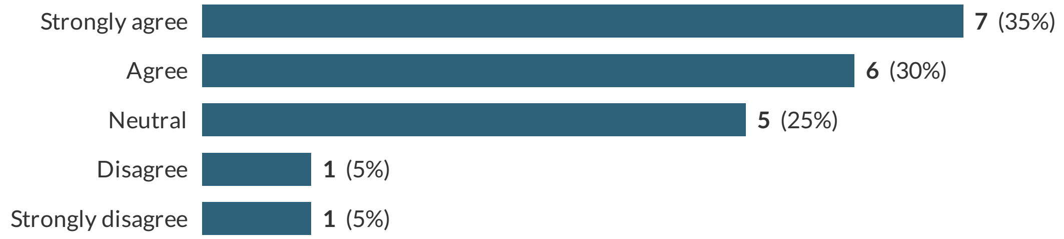

Multi answer: Percentage of respondents who selected each answer option (e.g. 100% would represent that all this question's respondents chose that option)

6.2 TBL is more effective for my learning than PBL

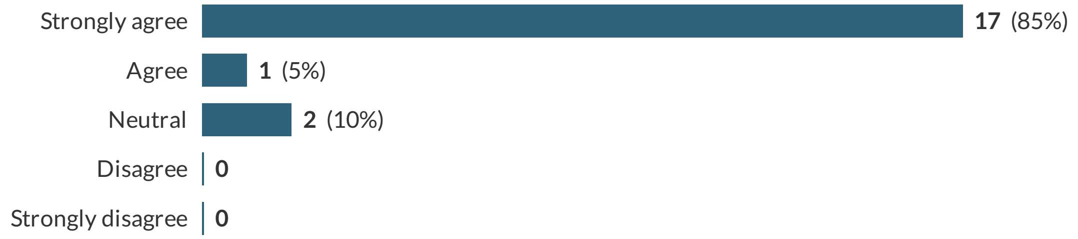

Multi answer: Percentage of respondents who selected each answer option (e.g. 100% would represent that all this question's respondents chose that option)

7 Overall experience. Please indicate your level of agreement

7.1 TBL is an effective learning method

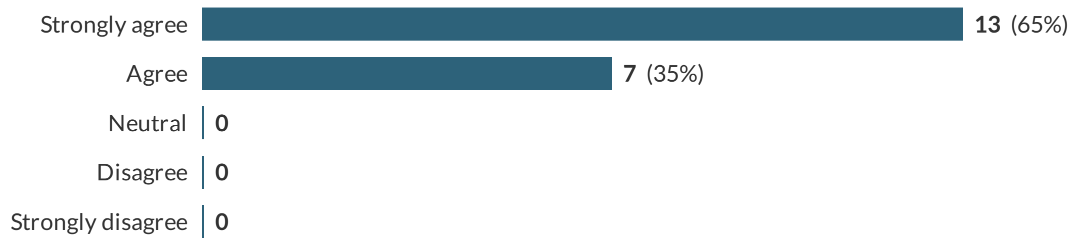

Multi answer: Percentage of respondents who selected each answer option (e.g. 100% would represent that all this question's respondents chose that option)

7.2 TBL should be used for all my teaching where possible

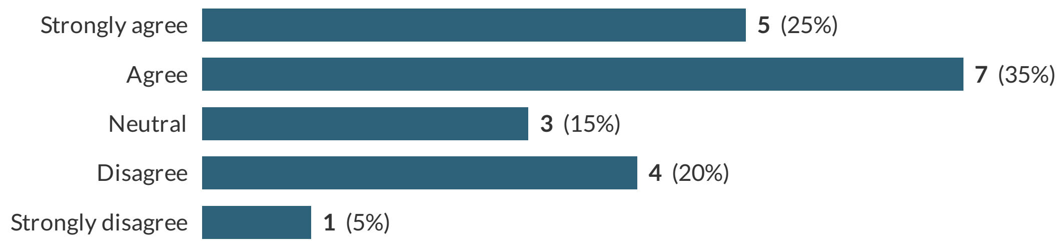

Multi answer: Percentage of respondents who selected each answer option (e.g. 100% would represent that all this question's respondents chose that option)

7.3 TBL should replace lectures

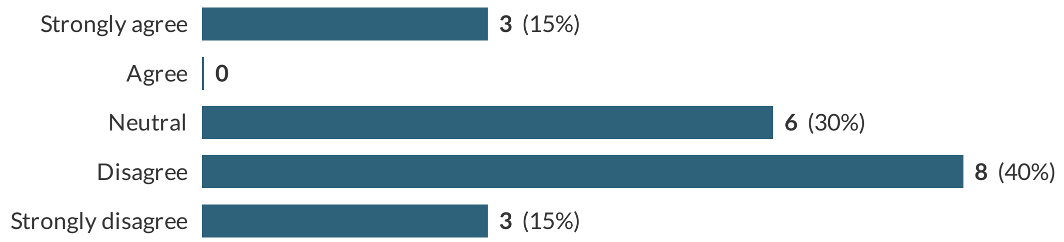

Multi answer: Percentage of respondents who selected each answer option (e.g. 100% would represent that all this question's respondents chose that option)

7.4 TBL should replace PBL

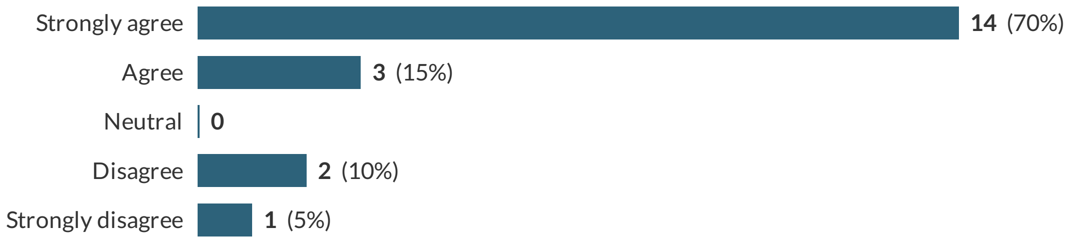

Multi answer: Percentage of respondents who selected each answer option (e.g. 100% would represent that all this question's respondents chose that option)

7.5 TBL should be used alongside lectures and PBL

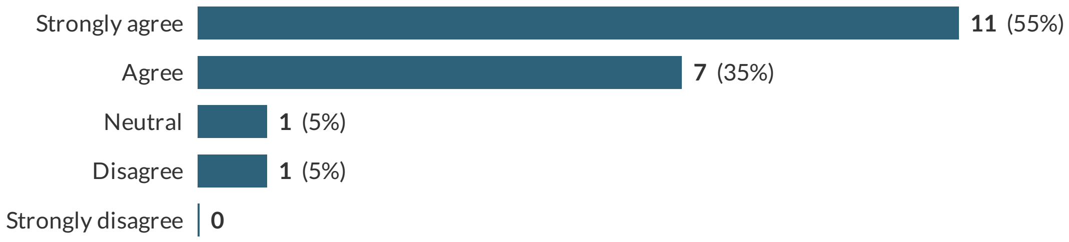

Multi answer: Percentage of respondents who selected each answer option (e.g. 100% would represent that all this question's respondents chose that option)

7.a Please comment on your answer

**Showing first 5 of 13 responses**	
For topics that are quite heavy such as chest pains, COPD, asthma, endocrine - thyroid, diabetes, TBL would be helpful to learn information	1082499-1082481-114082224
No PBL, time can be used better rather than PBL.	1082499-1082481-114082208
it should be used alongside lectures to supplement learning but not necessarily replace lectures	1082499-1082481-114082256
TBL is better than PBL.	1082499-1082481-114082346
PBL is more in depth and discussions are also carried out at the end of PBL so its very useful	1082499-1082481-114082281

---

8 Do you have any other comments?

Showing first 5 of 6 responses	
no	1082499-1082481-114082256
no	1082499-1082481-114082281
TBL is far most best method of interactive learning	1082499-1082481-114082227
If PBL was a problem to solve rather than a presentation on one condition it might be more beneficial.	1082499-1082481-114082207
N/A	1082499-1082481-114082183
